# PDGFRα: Expression and Function during Mitral Valve Morphogenesis

**DOI:** 10.3390/jcdd8030028

**Published:** 2021-03-13

**Authors:** Kelsey Moore, Diana Fulmer, Lilong Guo, Natalie Koren, Janiece Glover, Reece Moore, Cortney Gensemer, Tyler Beck, Jordan Morningstar, Rebecca Stairley, Russell A. Norris

**Affiliations:** Department of Regenerative Medicine and Cell Biology, Medical University of South Carolina, Suite 601 Basic Science Building, 173 Ashley Avenue, Charleston, SC 29425, USA; moorkels@musc.edu (K.M.); fulmerd@musc.edu (D.F.); guol@musc.edu (L.G.); korenn@musc.edu (N.K.); gloverja@musc.edu (J.G.); moorere@musc.edu (R.M.); gensemer@musc.edu (C.G.); beckt@musc.edu (T.B.); morningj@musc.edu (J.M.); stairley@musc.edu (R.S.)

**Keywords:** PDGFRα, mitral valve, endothelial, ERK1/2, cardiac development

## Abstract

Mitral valve prolapse (MVP) is a common form of valve disease and can lead to serious secondary complications. The recent identification of MVP causal mutations in primary cilia-related genes has prompted the investigation of cilia-mediated mechanisms of disease inception. Here, we investigate the role of platelet-derived growth factor receptor-alpha (PDGFRα), a receptor known to be present on the primary cilium, during valve development using genetically modified mice, biochemical assays, and high-resolution microscopy. While PDGFRα is expressed throughout the ciliated valve interstitium early in development, its expression becomes restricted on the valve endocardium by birth and through adulthood. Conditional ablation of *Pdgfra* with Nfatc1-enhancer Cre led to significantly enlarged and hypercellular anterior leaflets with disrupted endothelial adhesions, activated ERK1/2, and a dysregulated extracellular matrix. In vitro culture experiments confirmed a role in suppressing ERK1/2 activation while promoting AKT phosphorylation. These data suggest that PDGFRα functions to suppress mesenchymal transformation and disease phenotypes by stabilizing the valve endocardium through an AKT/ERK pathway.

## 1. Introduction

Mitral valve prolapse (MVP) is a common form of valve disease affecting 2–3% of the human population [1]. Nearly 1 in 10 MVP patients will require surgery and it is the fastest growing cardiovascular intervention in the Western world [2]. Mitral valve prolapse is characterized by the billowing of one or both leaflets beyond the annulus and into the left atrium as the result of structural changes that render the valve biomechanically incompetent during ventricular systole [3]. Oftentimes, prolapse is accompanied by improper leaflet closure leading to mitral regurgitation and secondary complications such as arrythmias, syncope, heart failure and sudden cardiac death. 

Recent genetic studies have identified several causal genes in patients with non-syndromic MVP. Mouse models generated with these corresponding mutations have revealed a developmental basis for MVP and have established various signaling mechanisms including primary cilia [4,5,6,7]. Primary cilia are cellular antennae that protrude from the cell and as such, are able to respond to various extracellular signals including growth factors, extracellular matrix (ECM) components, and blood flow, among others. For example, recent reports have demonstrated that cilia mediated desert hedgehog signaling promotes the intercellular cytoskeletal organization of valve interstitial cells and proper valve morphogenesis [8]. Other signaling factors, including platelet derived growth factor (PDGF), have been implicated as functioning through the primary cilia in other contexts but studies of their function during valve morphogenesis has not yet been evaluated. 

The PDGF signaling pathway includes growth factor ligands, PDGF-A, B, C and D, and two membrane receptor isoforms, PDGFRα and PDGFRβ. Specific PDGF ligands form dimers, which bind and activate homo- or heterodimerization leading to autophosphorylation at various cytoplasmic domains [9]. For instance, PDGF-BB can bind to PDGFRαα, PDGFRαβ, or PDGFRββ [10], while PDGF-AA exclusively activates PDGFRαα [11]. Once activated, PDGFR dimers act as tyrosine kinases and can propagate several downstream cascades involving PI3K, PLC-γ, and/or MAPK [9]. Unsurprisingly, both PDGFRα and PDGFRβ have been described in a plethora of cell functions including proliferation and survival [12], migration [13], and extracellular matrix production [14]. While many similarities exist between the isoforms, there is some evidence of compartmentalization within cells and tissues. 

Intriguing to mitral valve development, PDGFRα localizes to the ciliary axoneme in various cell types including cardiac cells [15] and is regulated by the intraflagellar transport (IFT) complex and other cilia proteins independently of PDGFRβ [16]. Further, *Pdgfra* depletion results in embryonic lethality with an array of developmental defects in mice [17] that are consistent with ciliopathies and include formation of the cardiac outflow tract [18], heart tube assembly, and cardiac fusion [19]. These data highlight the potential interplay between primary cilia and PDGF signaling during cardiac valve morphogenesis. 

Here, we describe the expression profile and consequences of PDGFRα loss of function that suggest a unique role for PDGFRα in the developing mitral valve. We show that both PDGF ligand and receptor proteins are temporally and spatially regulated during embryonic and postnatal valve morphogenesis. Loss of *Pdgfra* in a subset of endocardial cells resulted in disrupted intercellular junctions and an enlarged, myxomatous mitral valve phenotype at birth. These data suggest that there is likely a cilia-independent role for PDGFRα in stabilizing the valve endocardium after EMT. Additionally, these studies demonstrate that alterations of endocardial signaling is sufficient to engender a myxomatous valve phenotype consistent with observations of MVP patients. 

## 2. Materials and Methods

### 2.1. Mouse Husbandry and Genotyping

Animals were housed in a 12-h light-dark cycle with water and food ad libitum. *Pdgfra^fl/fl^* mice were obtained from Jackson Laboratories (B6.Cg-*Pdgfra^tm8Sor^*/EiJ, #006492). Conditional knockout animals were generated using previously described Nfatc1 Cre (*Nfatc1^Cre^)* or Nfatc1-enhancer Cre (*Nfatc1^enCre^)* [20] and genotyping was performed by Transnetyx, Inc. All animal experiments were performed under protocols approved by the Institutional Animal Care and Use Committees at the Medical University of South Carolina (protocol #2020-00956). Before cardiac resection, mice were euthanized by decapitation (embryonic stages) or isoflurane (Piramal) induction, followed by cervical dislocation in accordance with the Guide for the Care and Use of Laboratory Animals (NIH publication no. 85–23, revised 1996). Comparisons of the data generated for both male and female sexes showed no appreciable differences. As such, combined data for both sexes are shown.

### 2.2. Immunohistochemistry Staining and Imaging

Immunohistochemical staining (IHC) was performed on 5 μm sections of paraformaldehyde fixed, paraffin embedded E10.5, E13.5, E17.5, P0, and 4-month-old wildtype and/or *Pdgfra^f/f^* mouse hearts as previously described [4]. Sections of 15 μm thickness were used in cilia stains for 3D-IHC reconstructions. The primary antibodies and dilutions used were as follows: acetylated tubulin (Sigma, T6793, 1:500), α-smooth muscle actin (Sigma, A2547-100, 1:500), Collagen I (a gift from Dr. Stanley Hoffman, 1:250), GFP (Abcam, ab13970, 1:500), Ki67 (Abcam, ab15588, 1:100), MF20-c (Developmental Studies Hybridoma Bank, 1:50), NFATC1 (Invitrogen, MA3-024, 1:100), PECAM1/CD31 (Dianova, DIA-310, 1:50), PDGFRα (R&D, AF1062, 1:50), phospho-PDGFRα (Y754, Abcam, ab5460, 1:100), phosphorylated-p44/42 (Cell Signaling, 4370S, 1:50), vimentin (Epitomics, 2707-1, 1:400), and versican (a gift from Dr. Stanley Hoffman, 1:250). PDGFRα antibody exhibited no detectable staining in the conditional knockout mouse confirming its specificity (Figure S1). Secondary antibodies conjugated with AlexaFluor (488, 568, or Cy5) were purchased from Invitrogen (Carlsbad, CA, USA) and used at 1:100 dilution in 1% bovine serum albumin in PBS. Nuclei were stained with Hoechst (Life Technologies, #H3569, 1:10,000) and slides were cover-slipped with SlowFade Gold antifade reagent (Invitrogen, # S36936). IHCs were performed on at least 3 animals per genotype unless stated otherwise. Images were acquired with either Zeiss Axioscope (Zeiss, Inc., Oberkochen, Germany) or M2 Leica TCS SP5 AOBS Confocal Microscope (Leica, Inc. Wetzlar, Germany) equipped with LAS AF v2.6.3 Build 8173 Acquisition and Analysis Software. 

### 2.3. Three-dimensional Valve Reconstructions

For each animal, all sections containing valve were stained with hematoxylin and eosin (H&E), imaged with an Olympus BX40, aligned in Fiji, and traced to create 3D reconstructions in Imaris 9.3 software. Volume measurements of each leaflet (anterior and posterior) were obtained and normalized to total sections, *n* = 3–4 per genotype. 

### 2.4. Image Analyses

Counting of total, Ki67, and p-ERK1/2-positive cells was performed with CellProfiler with cropped images containing only the anterior leaflet; pipeline included the “IdentifyPrimaryObject” module for each channel of interest to generate counts and were compared in GraphPad Prism. Endothelial cells with positive PECAM1 and Ki67 staining were manually counted and substracted from total cell counts to segregate interstitial, total, and endothelial cell populations composing each leaflet. Proliferation studies were performed on 5 sections per animal in 3 animals per genotype. 

To quantify ECM differences, the channels of interest (versican or collagen I) were isolated and converted to grayscale in Adobe Photoshop. Integrated Density measurements were obtained using a fixed rectangle along the base, middle, and tip regions of each leaflet in ImageJ and analyzed in GraphPad Prism. 

### 2.5. Human Valve Endothelial Cell Culture and Treatments

Human valve endothelial cells (a kind gift from Dr. Joyce Bischoff) were cultured in endothelial cell growth media (PromoCell) on 1% Gelatin coated tissue culture plates (Corning). Cells were serum starved for 2 h, treated with PDGFR inhibitor, tyrphostin AG-1296 (Selleckchem, #S8024) or vehicle (dimethyl sulfoxide, Sigma Aldrich, #D2650) at a concentration of 2 μM and stimulated with PDGF-AA ligand at 50 ng/mL for 15 min (R&D Systems, #221-AA-025). 

### 2.6. Western Blotting

Protein was harvested using 1X RIPA, prepared with 2X SDS Laemmli buffer (125mM Tris pH7, 10% glycerol, 2% SDS, 5% β-mercaptoethanol, and 0.05% bromophenol blue) and separated via Bio-Rad mini blot gel electrophoresis chamber with 4–20% Mini-PROTEAN TGX Stain-Free Protein Gels (Bio-Rad, #456–8093). Protein was transferred to Trans-Blot Turbo Mini Nitrocellulose Transfer Packs (Bio-Rad, #170–4158) with Bio-Rad Trans-Blot Turbo semi-dry transfer system and blocked with 5% dry fat milk diluted in 1X TBS- 1% Tween (TBS-T). Membranes were incubated in primary antibodies diluted in 5% block (1:1000) overnight at 4 °C and included: p-AKT (#4060S), p-ERK1/2 (#4370S), AKT (#2920S) and ERK1/2 (#4695S) (Cell Signaling). Following washes in 1X TBS-T, blots were probed with anti-rabbit IgG HRP antibody (Sigma, #A9169, 1:7500) in 5% block for 1 h at room temperature, washed with 1X TBS-T, and exposed to West Femto maximum sensitivity substrate (ThermoFisher, Waltham, MA, USA, #34096) or Pierce ECL Western blotting substrate (ThermoFisher, #32209) prior to imaging on a Bio-Rad ChemiDoc MP Imaging System. Relative Densities of bands were measured in Fiji, normalized, and compared in GraphPad Prism. 

### 2.7. In Situ Hybridization

RNA in situ hybridization images for *Pdgfra* at E14.5 were obtained through GenePaint.org set ID: EG1832 and MH1215 [21] (Figure S4). Two riboprobes were generated. A 851 bp riboprobe was generated against region 2169–3019 of accession#: NM_001347719.1 A second riboprobe was generated against region 3147–3593 of accession#: M84607.1. Both probes analyze *Pdgfra* RNA expression at E14.5 and resulted in similar staining.

### 2.8. Statistical Analysis

All graphs and analyses were generated with GraphPad Prism 8.4.2. Statistical significance between genotypes or treatment groups were determined with a Student’s *t*-test or a one-way ANOVA with Tukey’s multiple comparisons when experiments involved more than two groups. Graphs include each data point plotted against group mean and error bars depicting standard error of mean (SEM). *n* = 3 per treatment or genotype group unless stated otherwise.

## 3. Results

### 3.1. PDGFRα Is Spatially and Temporally Regulated in Developing Mitral Valves

**Figure 1:**
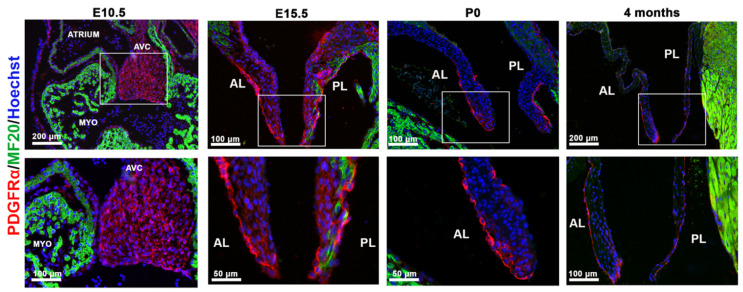
Spatiotemporal expression of PDGFRα during valve development. IHC of PDGFRα (red), myocardium (MF20, green) and nuclei (blue) showed expression of PDGF-receptor alpha protein throughout the valve interstitium early in development (E10.5) and is restricted to the endocardium through adulthood.

To characterize the expression profile of PDGFRα signaling during mitral valve development, we performed immunofluorescent staining on wildtype mouse mitral valve tissues from embryonic day 10.5 (E10.5) through adult time points. At E10.5, PDGF-receptor alpha (PDGFRα) is expressed on the membranes of nearly all valve endothelial and interstitial cells (Figure 1). Expression in the interstitium decreases at E15.5 and the majority of expression is observed on the valve endocardium. By postnatal day zero (P0), PDGFRα expression is mostly restricted to the membrane of endothelial cells lining the fibrosa region of both the anterior and posterior leaflets, a profile that is maintained through adulthood (Figure 1). 

**Figure 2:**
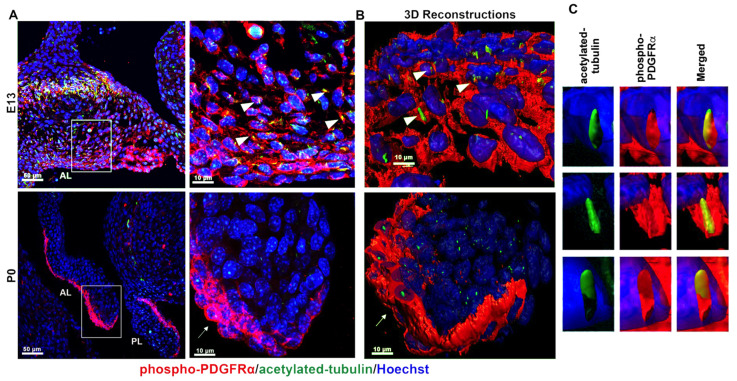
PDGFRα and ciliary axonemes early in development. (**A**) High-resolution microscopy and (**B**,**C**) 3D IHC reconstructions of 15 µm wildtype tissue show phosphorylated PDGFRα (red) present along ciliary axonemes (acetylated tubulin, green) (arrowheads highlighted in **C**) at E13. By P0, the receptor localizes to the endocardium where cilia are absent (arrows).

To test whether the PDGF-receptor could be activated in the valve, we probed for PDGFRα specific ligand, PDGF-A, and observed expression along cell membranes throughout the valve mesenchyme and endothelium at E13.5 and P0 (Figure S2). Phosphorylation of PDGFRα at tyrosine residue 754 (Y754) is known to be activated at the primary cilium in fibroblasts in response to PDGF-AA ligand binding. To determine whether this was occurring within the valve interstitium in vivo, we performed IHC with an antibody specific to phosphorylated Y754 on PDGFRα. At E13.5, when cilia are long and abundant [4], we observed activated PDGFRα broadly expressed on the cell membrane. A subset of this expression appeared to localize with acetylated alpha tubulin (Figure 2A). Three-dimensional reconstructions of IHCs further showed the presence of activated PDGFRα along the ciliary membranes as well as more broadly present within the valve interstitial cells and along the cell membrane (Figure 2B). By P0 and consistent with previously reports [4], cilia are scant within the mitral valve interstitium and activated PDGFRα becomes localized to the valve endothelium in cells devoid of primary cilia (Figure 2C,D). Together, these data suggest that while it is possible that PDGFRα functions through primary cilia early in development, it is likely functioning independently of primary cilia in the post-EMT valve endocardium. 

### 3.2. Genetic Ablation of Pdgfra in the Valve Endocardium Leads to Abnormal Valve Morphogenesis

**Figure 3:**
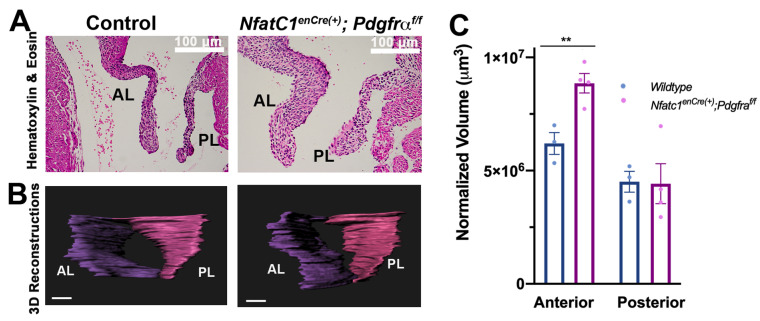
Genetic ablation of *Pdgfra* in the valve endocardium results in abnormal valve morphogenesis at P0. (**A**,**B**) Hematoxylin and eosin (H&E) staining and 3D reconstructions of P0 mitral valves depleted of *Pdgfra* with *Nfatc1^enCre^,* scale bars = 100 μm. (**C**) Quantification of normalized leaflet volumes measured by 3D reconstructed surfaces reveals significant thickening in *Nfatc1^enCre(+)^;Pdgfra^f//f^* anterior leaflets compared to control littermates. *n*= 3–4 per genotype, ** *p* < 0.005 with Student’s *t*-test.

The distinct expression profile of PDGFRα in the valve endothelium was reminiscent of previously described Nfatc1-enhancer Cre (*Nfatc1^enCre^*), which traces endocardial cells that do not undergo EMT [20]. Additionally, nuclear factor of activated T cells, cytoplasmic 1 (NFATC1) protein expression is robust in the endocardium by E11.5 and precedes endocardial PDGFRα expression until E14.5 when the receptor is diminished in the interstitium (Figure S3). This suggested that PDGFRα is present in a unique subset of valve endocardial cells that are also *NfatC1^enCre(+)^*. To test the function of PDGFRα in this unique cell population, conditional mice were generated and are designated as *NfatC1^enCre(+)^; Pdgfra^f/f^*. Depletion of *Pdgfra* in this subpopulation of valve endocardial cells led to significant valve thickening by P0 compared to wildtype control littermates (Figure 3F–H). Interestingly, the phenotypic change in valve volume was only observed in the anterior leaflet.

### 3.3. Perturbation of Pdgfra Results in Hyperplastic Valves, Altered Downstream Signaling and Changes in ECM

**Figure 4:**
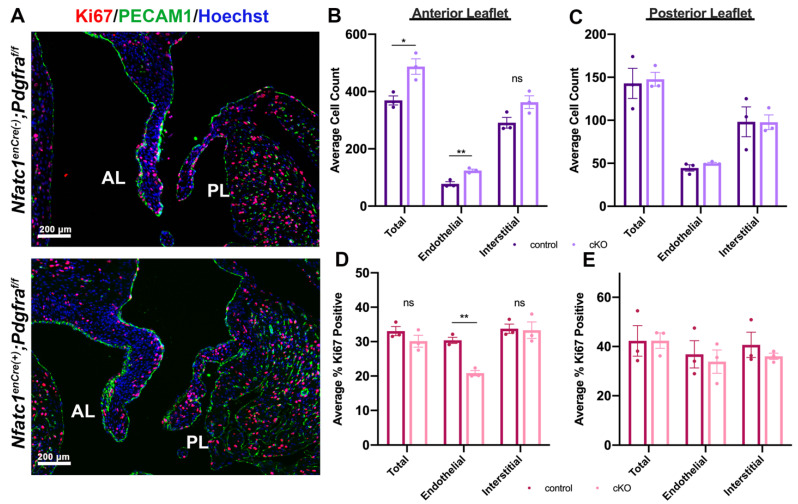
*Pdgfra* depleted leaflets contain more cells at P0. (**A**) IHC of proliferating cells (Ki67 = red), endothelial cells (PECAM1 = green) and nuclei (blue) of *Nfatc1^enCre(+)^;Pdgfra^f/f^* and littermate control mice at postnatal day zero (P0). (**B**,**C**) Quantification of total nuclei counted in the endothelium and interstitium of each leaflet shows increased total and endothelial cell populations in *Nfatc1^enCre(+)^;Pdgfra^f/f^* (conditional knockout, cKO) anterior leaflets compared to littermate controls. Quantification of Ki67 positive cells within the anterior (**D**) and posterior (**E**) leaflets shows decreased endothelial cell proliferation of cKO compared to control anterior leaflets. Total and proliferating cells were counted in 4–5 sections per animal, 3 animals per genotype, * *p* < 0.05 and ** *p* < 0.005 with Student’s *t*-test, ns = not significant.

As a potential explanation of increased valve size, total cell counts and proliferation indices were determined. Concomitant with 3D morphometrics, counting of total nuclei within the *Nfatc1^enCre(+)^;Pdgfra^f/f^* compared to control littermate valves revealed a statistically significant increase in total and endothelial cells of anterior leaflets and no significant differences were observed in the posterior leaflets. No global proliferation effects were observed as measured by Ki67 positive cells. However, when categorized by cell type, a significant decrease in percentage of proliferating cells was observed in endothelial cells of *Nfatc1^enCre(+)^;Pdgfra^f/f^* anterior leaflets compared to controls (Figure 4). 

**Figure 5:**
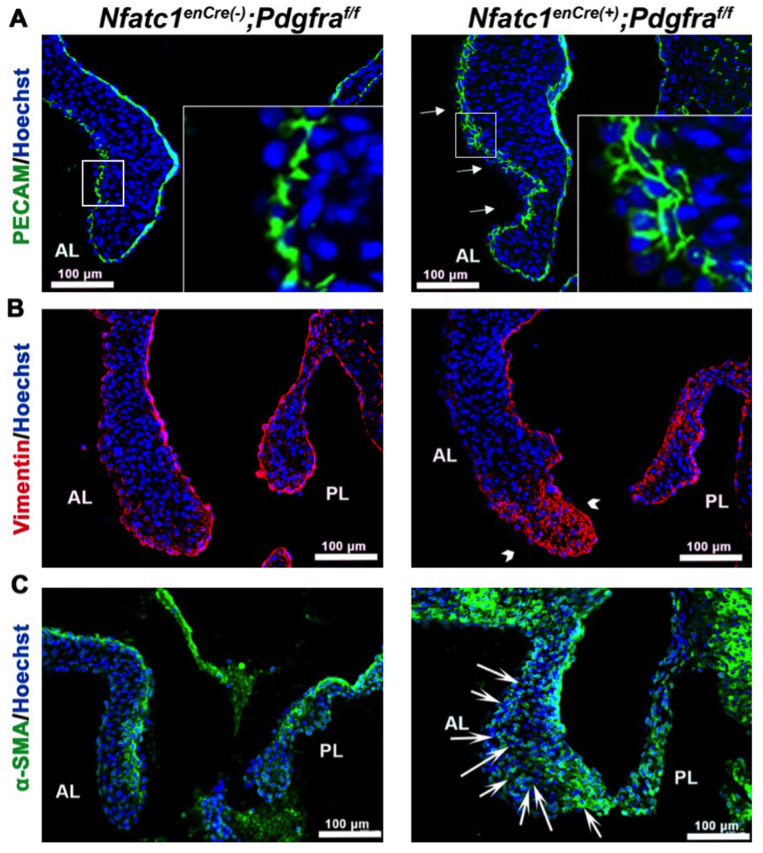
*Pdgfra* depletion in the valve endocardium leads to disrupted endothelium and expression of myofibroblast markers in the valve interstitium. (**A**) IHC of PECAM1 (green) shows disrupted endothelial adhesions along the fibrosa of *Nfatc1^enCre(+)^;Pdgfra^f/f^* anterior leaflets (arrows). (**B**,**C**) IHC of vimentin (red) and alpha-smooth muscle actin (α-SMA, green) exhibits increased expression of vimentin in the tip region (arrowhead) and alpha smooth muscle actin throughout the valve interstitium (arrows) in *Nfatc1^enCre(+)^;Pdgfra^f/f^* mice compared to controls.

Given the robust expression of PDGFRα in the endothelium, concomitant with increased cell number in the conditional knockouts, we hypothesized that loss of PDGFRα may result in a destabilized endothelium and increased endothelial to mesenchyme transition (EMT or EndoMT). An initial step of EMT is the destabilization of adherens junctions allowing the disassociation of cells from the endothelial layer to migrate into the basement membrane and surrounding extracellular matrix (ECM). IHC staining for PECAM1 (platelet and endothelial cell adhesion molecule 1) revealed uneven staining along the fibrosa of *Nfatc1^enCre(+)^;Pdgfra^f/f^* valve leaflets compared to controls (Figure 5A). The staining of PECAM1 in the conditional knockout is consistent with cell polarity defects that can be attributed to altered cell–cell functions [22]. Additionally, knockout valves exhibited enhanced expression of vimentin and α-smooth muscle actin, markers that have been shown to be upregulated in diseased valves and in valve endothelial cells with re-activated EMT. Expression of both of these markers were ectopically expressed in the tip and in regions proximal to the fibrosa of the mitral leaflets, corresponding with the localization of PDGFRα (Figure 5B,C).

**Figure 6:**
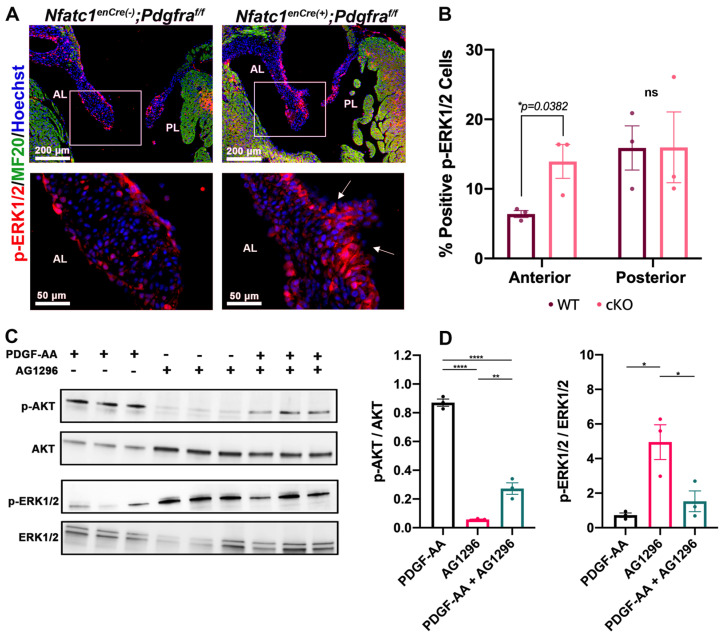
*Pdgfra* inhibition leads to increased phosphorylated-ERK1/2 expression. (**A**) IHC for phosphorylated-ERK1/2 (red), MF20 (green) and nuclei (blue) shows increased p-ERK1/2 in the tip region of *Nfatc1^enCre(+)^;Pdgfra^f/f^* anterior leaflets (AL) compared to wildtype controls (arrows) at P0. (**B**) Quantification of p-ERK1/2 positive cells in wildtype and *Nfatc1^enCre(+)^;Pdgfra^f/f^* anterior and posterior leaflets analyzed with CellProfiler, * *p* < 0.05 with Student’s *t*-test, *n* = 3 per genotype. (**C**) Western blots of protein isolated from serum starved human valve endothelial cells treated with PDGFR inhibitor (Tyrphostin AG-1296, 2 μM for 45 min) or vehicle (DMSO) and stimulated with 50 ng/mL PDGF-AA for 15 min. (**D**) Quantification of the ratio of phosphorylated to total protein in each treatment, *n* = 3 per treatment, * *p* < 0.05, ** *p* < 0.005, **** *p* < 0.001 with a One-way Anova with Tukey’s multiple comparisons, ns = not significant.

To determine whether loss of *Pdgfra* in the valve endocardium results in altered downstream signaling, we initially performed IHC staining for phosphorylated ERK1/2 (p-ERK1/2). Surprisingly, we noted an increase in p-ERK1/2 levels at the tip of the anterior leaflets in *NfatC1^enCre(+)^;Pdgfra^f/f^* mice compared to wildtype controls (Figure 6A,B), suggesting that PDGFRα can function either directly or indirectly to suppress the activation of ERK1/2. This observation is contrary to previous studies showing that PDGFRα is required for ERK1/2 activation. This suggested the possibility that suppression of ERK1/2 may confer a unique tissue or cell-specific function of PDGFRα in MAPK signaling. To test this possibility, we stimulated primary human valve endothelial cells with PDGF-AA ligand in the presence or absence of the PDGFRα inhibitor, Tyrphostin AG1296. Similar to our in vivo results, Western blot analyses from these cultures revealed that supplement of PDGF-AA ligand to valve endothelial cells resulted in a loss of active ERK1/2 signaling. Similarly, PDGF receptor blockade with Tyrphostin AG-1296 resulted in significant activation of p-ERK1/2 (Figure 6C,D). As PDGFRα is also known to stimulate AKT through PI3K, we assayed whether PDGF-AA ligand could alter activation patterns of this signaling pathway. Western analyses revealed that, unlike ERK1/2 activation, AKT is potently activated by PDGF-AA ligand, which is suppressed following addition of the receptor inhibitor, AG-1296 (Figure 6C,D). These data demonstrate that PDGF-AA ligand functions through PDGFRα to activate the AKT signaling pathway and suppress MAPK-ERK1/2 activation in vivo and in vitro.

**Figure 7:**
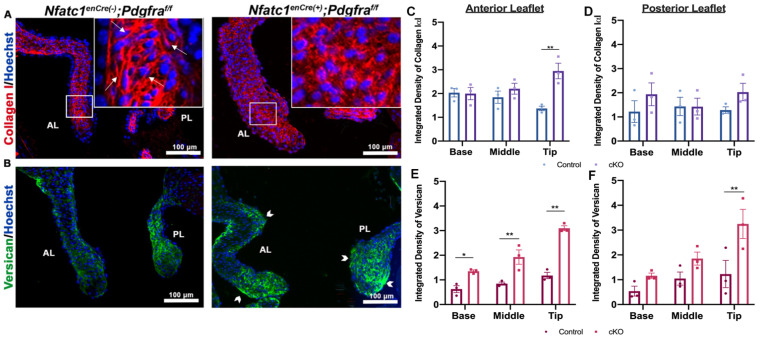
ECM components are disorganized in *Pdgfra* conditional knockouts at P0. (**A**,**B**) IHC of collagen (red) versican (green), and nuclei (blue) and (**C**–**F**) quantification of fluorescent intensity in based, middle, tip regions of the valve shows loss of fibrillar collagen (arrows) and increased versican expression (arrowheads) in *Nfatc1^enCre(+)^;Pdgfra^f/f^* knockouts compared to wildtype controls. *n* = 3 per genotype and * *p* < 0.05, ** *p* < 0.005 with Student’s *t*-test.

Activation of ERK1/2 has been associated with increased collagen and proteoglycan production, which are hallmarks of MVP patients with myxomatous degeneration. Thus, we evaluated whether there is an increase in these ECM proteins that correlate with ERK1/2 activation. As shown in Figure 7, there is a modest, but consistent increase in both collagen 1α1 and versican within the mitral leaflets of *NfatC1^enCre(+)^; Pdgfra^f/f^* valves compared to littermate controls. In the conditional knockout mice, collagen 1α1 expression appears throughout the valve proper and exhibits a grainy, non-fibrillar appearance compared to the control animals (Figure 7A) with significant increases in the tip region of the anterior leaflets (Figure 7C,D). There are also noted differences in versican expression between the genotypes. Versican expression is not only more abundant, particularly at the tip of the mitral valves, but also has increased expression on both the atrialis and fibrosa of the leaflet (Figure 7E,F). This finding of altered ECM organization, as evidenced by non-fibrillar collagen and expanding versican expression, is consistent with a myxomatous change in the valves observed in MVP patients. 

## 4. Discussion

Our study demonstrates that PDGFRα protein is expressed in the developing mitral valve and its deletion leads to abnormal valve morphogenesis indicative of early disease phenotypes. Expression of PDGFRα has mostly been described in fibroblastic mesenchyme cells and recent cardiac studies support its expression in neural crest, mesoderm, and epithelial cells [18,23,24]. Our data support previous studies that identified *Pdgfra* mRNA transcripts throughout the atrioventricular valve of developing mice [25] and demonstrated co-localization along ciliary axonemes in vivo [15]. While reports have exhibited co-localization of PDGFRα with vimentin in human ventricles [26] and in endothelial and endothelial derived mesenchyme cells [27], the robust endothelial expression of PDGFRα was unexpected. 

The unique localization of PDGFRα along the endocardium of the fibrosa led us to hypothesize a cilia-independent role in the valve endocardium. This hypothesis is in part due to our recent studies that demonstrated a lack of primary cilia along the valve endocardium [6]. While our studies have focused on the mitral valve, the regionalized expression of *Pdgfra* mRNA transcripts are similarly observed in all cardiac valves at E14.5 (Figure S4). The unilateral expression of PDGFRα on the fibrosa side of the mitral valve is consistent with a role in responding to flow rates or shear stress. In this context, areas of low shear stress have increased PDGFRα expression whereas high shear stress rates that are observed on the atrialis side of the mitral leaflets display no detectable PDGFRα protein or mRNA expression. These data suggest that expression of *Pdgfra* may be impacted by the valve’s mechanical environment and inhibited by high shear stress. If true, the regionalized localization of this receptor may confer a mechanically induced endocardial phenotype that helps specify the sides of the valve. This hypothesis is consistent with increasing reports that suggest the importance of biomechanical cues in developmental gene expression. For example, KLF2/KLF4 responds to shear stress on the atrial aspect of the valve and promotes transcriptional activation of Wnt family member 9B, *Wnt9b,* and establishment of a WNT signaling gradient within the valve [28]. In this context, KLF2/4 are induced by shear-stress whereas *Pdgfra* is suppressed. Therefore, WNT and PDGF gradients on opposing sides of the mitral leaflet may contribute unique, spatially oriented signaling gradients that result in establishment of valve layers during neonatal life. 

Given previous reports showing no atrioventricular valve phenotypes with *Mesp1^Cre^* or *Tie2^Cre^* [23,27] and a similar expression profile of the NFATC1 transcription factor, we decided to conditionally ablate *Pdgfra* with Nfatc1-enhancer Cre (*Nfatc1^enCre^)*, which targets endothelial cells that do not undergo EMT [20]. Unlike other MVP mouse models that exhibit enlarged leaflets as a result of ECM and cell compaction defects [4,5,8], we observed hypercellular leaflets in *Pdgfra* knockout mice. While it was intuitive to anticipate increased proliferation as a contributor, we observed decreased proliferation in the endothelial cells of knock-out anterior leaflets and no significant effects in other valve regions. Previous studies describe PDGFRα as promoting proliferation [12] and support our finding that loss of PDGFRα would disrupt proliferative capabilities. Future studies should evaluate this effect at earlier and more proliferative stages. Further, no changes in cell death or growth were observed in hypocellular aortic valves of *Tie2^Cre+^*;*Pdgfra* knockout mice [27], suggesting an alternative developmental process is at work in the context of *Pdgfra* depletion. The temporal expression of endogenous NFATC1 and PDGFRα may suggest direct regulation of PDGFRα expression by *Nfatc1*. Indeed, previous reports have identified putative NFATC1 elements within the proximal PDGFRα promoter [29]. Further studies are needed to validate this observation.

It is interesting to note that we observed valve phenotypes primarily associated with the anterior leaflet. This was a surprising finding given that PDGFRα is expressed in both the anterior and posterior mitral leaflets. Unlike the anterior leaflet, the posterior leaflet has contributions from epicardial-derived cells EPDCs, a cell type that expresses PDGFRα [30] (Figure S4). It is currently unknown whether this additional cell population has an influence on PDGFRα or enables compensation for the loss of this receptor on posterior leaflet endocardial cells. Nonetheless, our data suggest a functional difference between each leaflet in regulation and response of PDGFRα signaling.

During normal valvulogenesis, endocardial cells receive cues from the myocardium (i.e., TGFbeta and BMP), disassemble their adherens junctions, invade the proteoglycan rich valve cushion, and differentiate into mesenchymal interstitial cells in a complex process known as endothelial to mesenchyme transition (EndoMT or EMT) [31]. Interstitial cells then proliferate and converge to a specified density and align to form a stratified ECM. While a plethora of signaling pathways have been implicated in the regulation of EMT during valve development [32], NFATC1 is appreciated as a master of endothelial cell-fate decision making in both mice and zebrafish [20,33,34,35,36]. Specifically, loss of *Nfatc1* led to shortened and thicker valves as a result of increased endothelial cells populating the interstitium [20,37], suggesting that NFATC1 is required to suppress EMT in a subset of valve endothelial cells and to promote valve elongation. Cell–cell adhesion breaks are an additional indicator of endothelial instability and have implications in valve disease. Since EMT diminishes by E13, it is expected that adhesion complexes between endothelial cells remain intact throughout life. However, *Pdgfra* deletion led to abnormal PECAM1 organization and layers of PECAM1 positive interstitial cells similar to what is observed in diseased valves with prolonged EMT [38,39]. We also observed increases in α-smooth muscle actin and vimentin in the interstitium of knockout valves. These findings, combined with our observations of increased cell number in the absence of excess proliferation, may indicate increased EMT in the *Pdgfrα* conditional knockout, although future lineage tracing experiments are needed to add support to this finding. As illustrated by Anstine et. al., a major consequence of disrupted adhesions is the potential for circulating contents to diffuse through the endothelial layer [40]. It has also been shown that CD45+ macrophages and other inflammatory cells are able to invade the valve interstitium and may illicit further fibrotic responses [41,42,43]. Thus, defects in PDGFRα could lead to a disrupted endothelium and a progression of valve disease that is exacerbated by immune cell infiltration among other mechanisms.

Dysregulation of the valve ECM is another hallmark of valve disease and facilitates a unique fibrotic microenvironment. Given the stratification and organization of the valve tissue, it is interesting that PDGFRα expression is restricted to the fibrosa. As collagen is abundant in this region, future studies should investigate if matrix metallopeptidases (MMPs) and other ECM modulators are downstream of PDGFRα in the context of valve cell migration and EMT. As observed in diseased valves, we showed increases in collagen 1α1 and versican in regions proximal to PDGFRα+ endothelium. We noted that the collagen I expression in diseased valves appeared to be organized differently compared to the littermate controls. Activated ERK1/2, a known regulator of cardiac fibrosis that has been observed in various models of mitral valve disease [43,44] is also increased in *Pdgfra* depleted valves in similar regions and in valve endothelial cells treated with PDGFR inhibitor. It is tempting to speculate that the increase in p-ERK1/2, which is known to directly and indirectly regulate MMP2 transcription and activation, is intimately involved in extracellular matrix remodeling in the valve [43]. Thus, regulation of MMP2 by activated ERK1/2 could provide a mechanistic basis that contributes to the myxomatous phenotype observed in MVP patients. 

While we have not conclusively determined how *Pdgfra* depleted valves become enlarged, it is likely that increased total cell counts lead to greater ECM content and leaflet volume. Further, increased activation of p-ERK1/2 and alpha-smooth muscle actin are suggestive of increased myofibroblast activation, a cell state that often results in greater ECM deposition. The disorganization of versican and collagen may also be a result of perturbed cell–ECM interactions through mechanisms that have not yet been characterized within the mitral valve. Future studies are necessary to decipher the distinct PDGFRα regulation of EMT, cell–ECM organization and/or ECM production during earlier stages of valve development.

Taken together, this study supports a role for PDGFRα in regulating valve endocardial cell homeostasis. Future studies focused on methods for stabilizing the valve endocardium may be applicable as therapeutics for those with MVP.

## Data Availability

Raw data presented in this study are available on request from the corresponding author.

## Supplementary Materials

The following are available online at https://www.mdpi.com/2308-3425/8/3/28/s1, **Figure S1:** PDGFRα antibody validation, **Figure S2.** PDGF-A expression during mitral valve development, **Figure S3**. NFATC1 protein is expressed in the valve endothelium early in development, **Figure S4.** mRNA Expression of *Pdgfra* in the heart at E14.5.
