# Supplementary material for: PDGFRα: Expression and Function during Mitral Valve Morphogenesis
Source: J Cardiovasc Dev Dis. 2021 Mar 13;8(3):28. doi: 10.3390/jcdd8030028 (PMC7999759; doi:10.3390/jcdd8030028)
Supplement: Supplementary file 1 [file jcdd-08-00028-s001.pdf]

Supplemental Figures

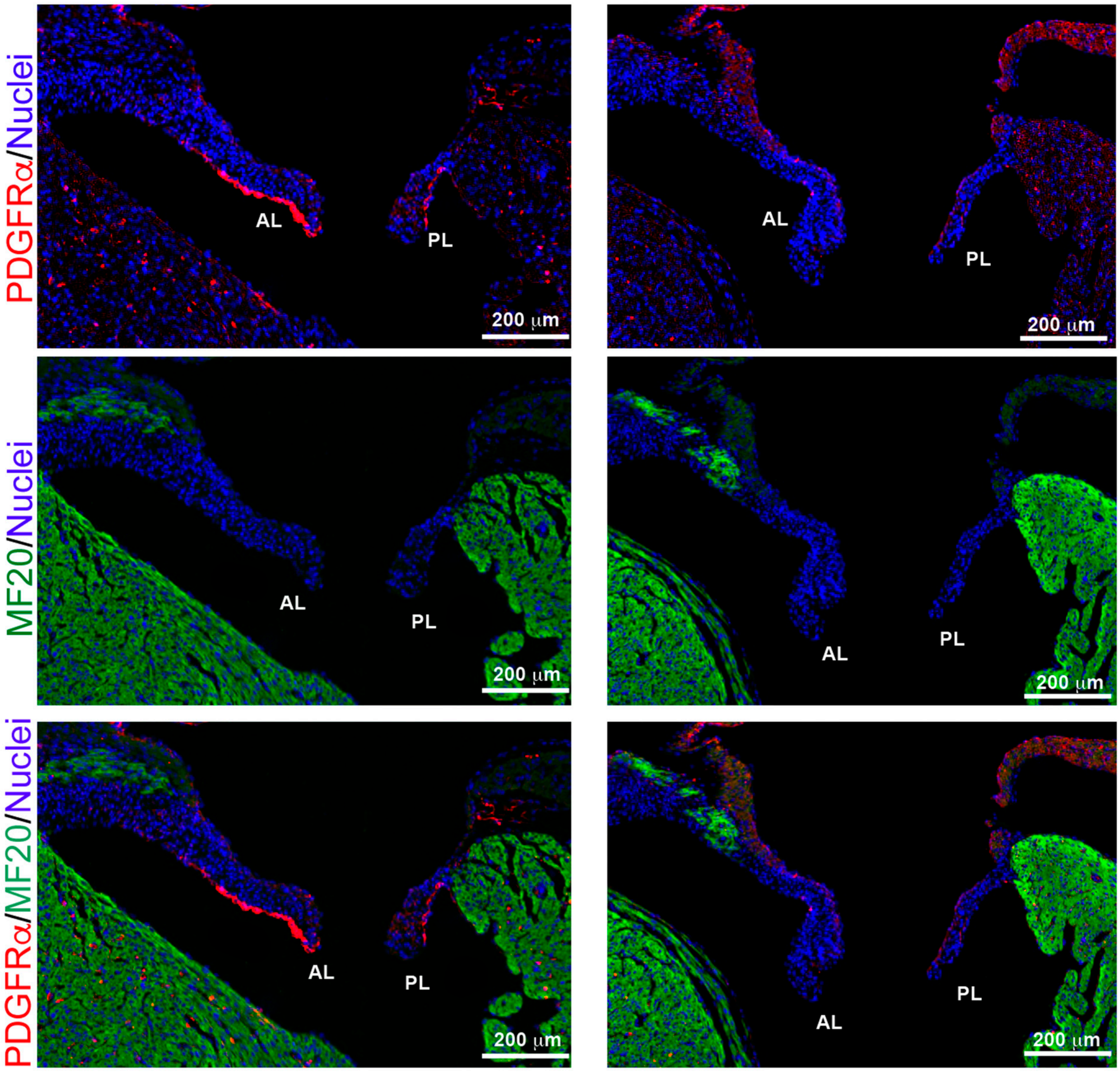

Figure S1. PDGFRα antibody validation. IHC of PDGFRα (red), myocardium (green) and nuclei (blue) in *Nfatc1*$^{enCre(+)}$;*Pdgfra*$^{f/f}$ mitral valves compared to wildtype control. Expression of PDGFRa is gone in the conditional knockout mitral valves while punctate staining remains in both the atrial appendage and left ventricular wall. These stains demonstrate specificity of both the antibody and Cre.

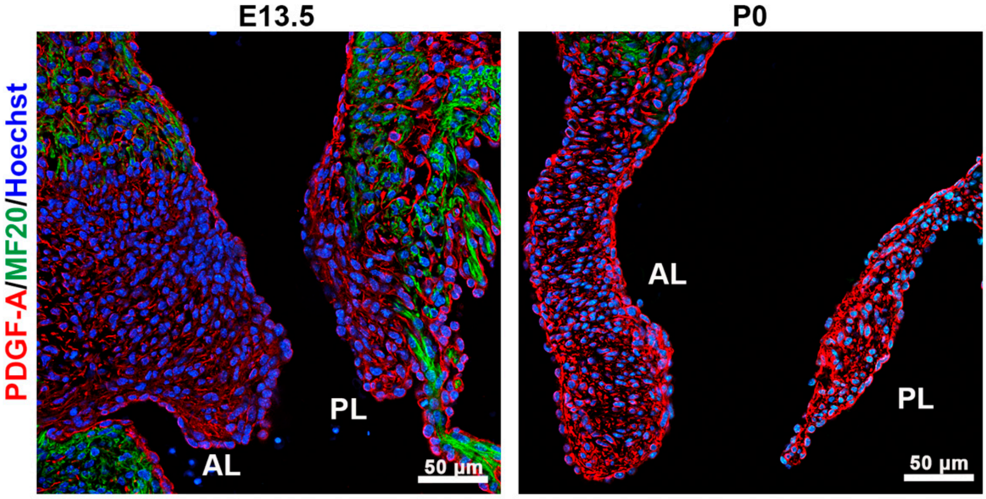

Figure S2. PDGF-A expression during mitral valve development. IHC of PDGF ligand, PDGF-A (red), MF20 (green) and nuclei (blue) in mitral leaflets of wildtype E13.5 and P0 mice.

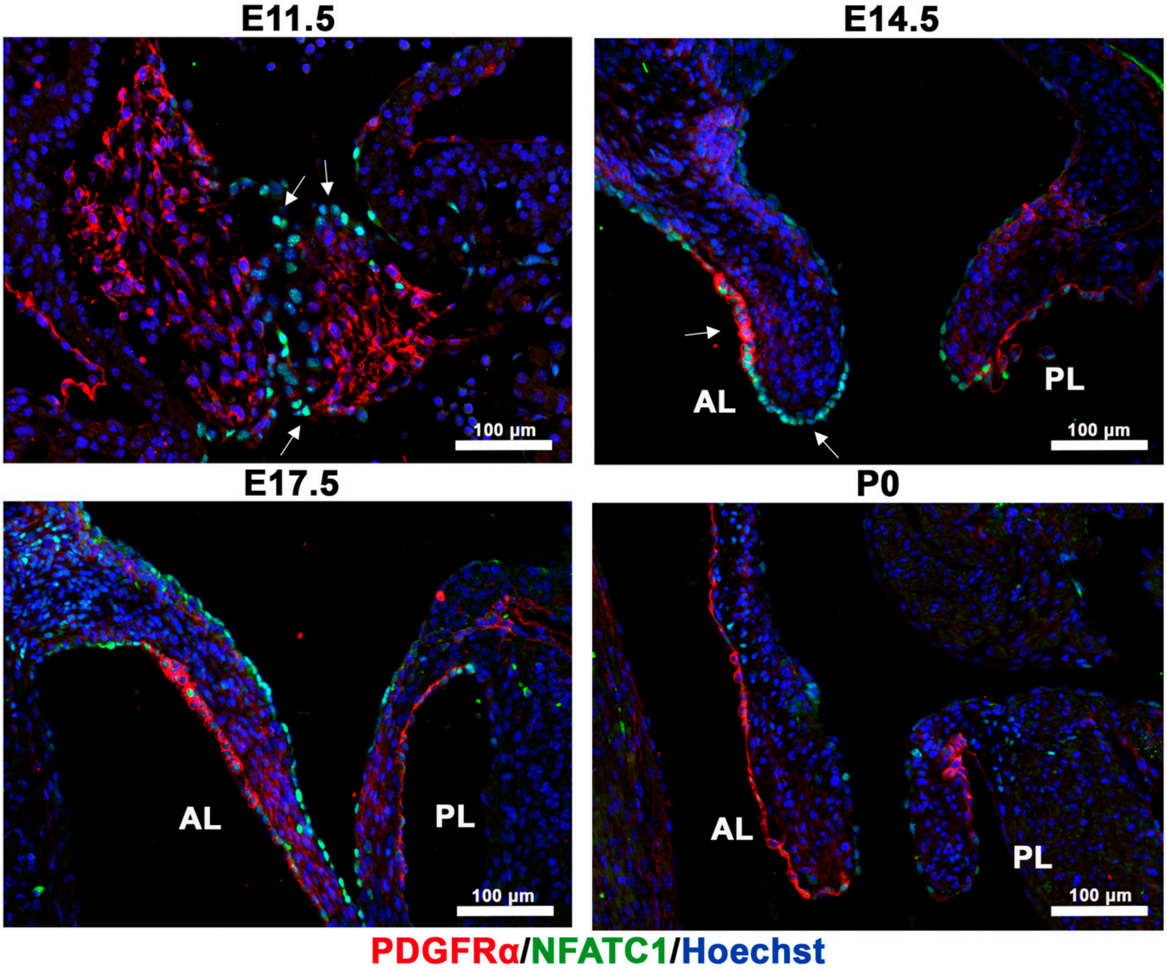

Figure S3. NFATC1 protein is expressed in the valve endothelium early in development. IHC of NFATC1 (green), PDGFRα (red), and nuclei (blue) in E11.5-P0 wildtype murine mitral valves. NFATC1 protein is expressed in the endocardial cells (arrows).

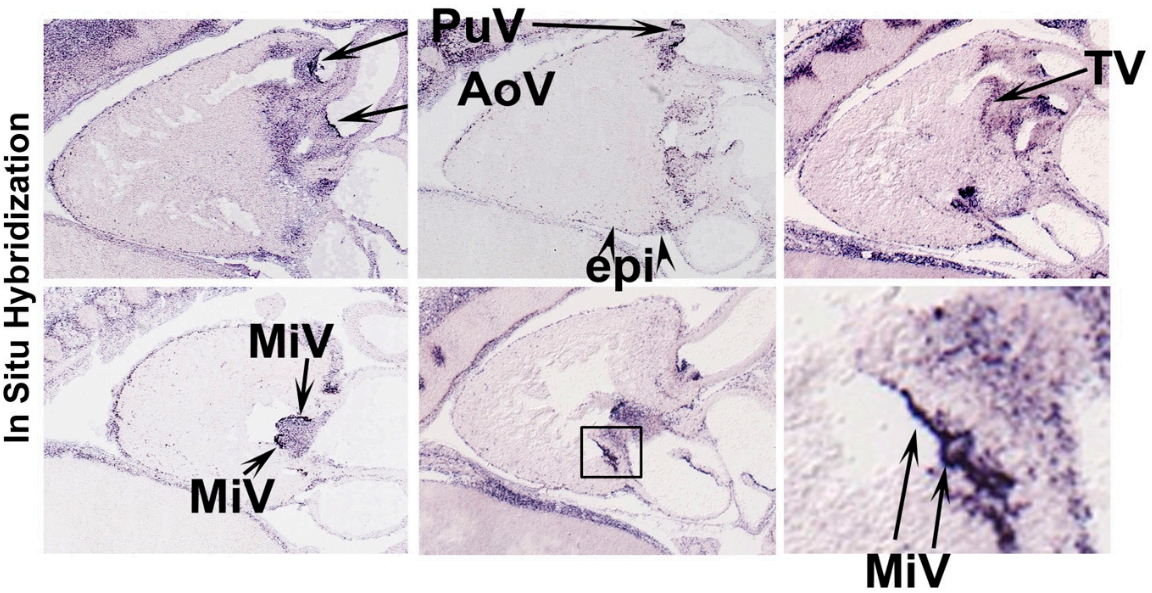

Figure S4. mRNA Expression of *Pdgfra* in the heart at E14.5. In situ hybridization of *Pdgfra* in wildtype E14.5 mouse embryos obtained from GenePaint.org set ID: EG1832 and MH1215. Expression of *Pdgfra* is observed in all valve leaflets including the pulmonary valve (PuV), aortic valve (AoV), tricuspid valve (TV) and mitral valve (MiV). In each of these valves expression is observed throughout the leaflet/cusps, but is more concentrated on the low flow fibrosa side. Expression is also evident within the epicardium (epi). Expression within the ventricle is much lower or undetectable compared to valve structures.
